# Gelechiidae Moths Are Capable of Chemically Dissolving the Pollen of Their Host Plants: First Documented Sporopollenin Breakdown by an Animal

**DOI:** 10.1371/journal.pone.0019219

**Published:** 2011-04-28

**Authors:** Shixiao Luo, Yongquan Li, Shi Chen, Dianxiang Zhang, Susanne S. Renner

**Affiliations:** 1 Key Laboratory of Plant Resource Conservation and Sustainable Utilization, South China Botanical Garden, The Chinese Academy of Sciences, Guangzhou, China; 2 Department of Biology, University of Munich (LMU), Munich, Germany; University of Melbourne, Australia

## Abstract

**Background:**

Many insects feed on pollen surface lipids and contents accessible through the germination pores. Pollen walls, however, are not broken down because they consist of sporopollenin and are highly resistant to physical and enzymatic damage. Here we report that certain Microlepidoptera chemically dissolve pollen grains with exudates from their mouthparts.

**Methodology/Principal Findings:**

Field observations and experiments in tropical China revealed that two species of *Deltophora* (Gelechioidea) are the exclusive pollinators of two species of *Phyllanthus* (Phyllanthaceae) on which their larvae develop and from which the adults take pollen and nectar. DNA sequences placed the moths and plants phylogenetically and confirmed that larvae were those of the pollinating moths; molecular clock dating suggests that the moth clade is younger than the plant clade. Captive moths with pollen on their mouthparts after 2-3 days of starvation no longer carried intact grains, and SEM photographs showed exine fragments on their proboscises. GC-MS revealed cis-β-ocimene as the dominant volatile in leaves and flowers, but GC-MS analyses of proboscis extracts failed to reveal an obvious sporopollenin-dissolving compound. A candidate is ethanolamine, which occurs in insect hemolymphs and is used to dissolve sporopollenin by palynologists.

**Conclusions/Significance:**

This is the first report of any insect and indeed any animal chemically dissolving pollen.

## Introduction

Obligate mutualisms are often supported by unique adaptations that facilitate specific interactions between the partners. Well-known examples are found among plants and their pollinators, and a striking number of the most tightly co-evolved interactions involve moths. Examples include yucca flowers and yucca moths [1], senita cacti and senita moths [2], and Phyllantheae flowers and *Epicephala* moths [3], [4]. In each of these mutualisms, special behaviors or mouthparts evolved, resulting in efficient transport of pollen to stigmas. None of these moths feed on pollen. Moths that actively ingest pollen belong to relatively old families with biting-chewing mouthparts capable of crushing the walls of spores or pollen grains, such as Agathiphagidae, Heterobathmiidae, and Micropterigidae [5], [6], [7]. Higher Lepidoptera instead have mouthparts adapted for fluid feeding, and none are known to regularly ingest pollen except species of *Heliconius* (including *Laparus*), which digest pollen contents (but not pollen walls) in a mix of saliva and nectar [8], [9], [10].

The reason Lepidoptera and other insects, including bee larvae who obligately feed on pollen, all void the walls of pollen and spores intact is that these walls consist of sporopollenin, a complex polymer that is composed of fatty acids and phenolic compounds. The precise structure of sporopollenin is unknown [11]. Sporopollenin is resistant to nonoxidative physical, chemical, and biological treatments and insoluble in both aqueous and organic solvents [12], [13]. This chemical inertness accounts for the preservation of pollen grains for tens to hundreds of millions of years [14]. There is also an extensive fossil record of insects gut contents containing pristine pollen [15]. Yet spores and pollen contain up to 61% protein [16] and are an important food source for many animals whose gut enzymes enable them to digest substances on pollen surfaces, such as pollenkitt, as well as pollen contents, accessible through the spores' germination pores.

Here, we report that certain gelechiid moths in the genus *Deltophora* are capable of chemically dissolving the pollen walls' sporopollenin. This is the first record of the chemical breakdown of spore walls by any eukaryote. The *Deltophora* moths have highly specialized proboscises (c. 3 mm long) and are the only pollinators of two unrelated species of *Phyllanthus* in the Phyllanthaceae, a family comprising 1750 species in 59 genera (1270 in *Phyllanthus*), several with intricate phenology and pollinator interactions [4], [17]. We carried out field observations on the *Deltophora*/*Phyllanthus* mutualism at several sites in tropical China, with one population studied over four years to document the phenological fit between the partner's life cycles and to verify that the plants had no other pollinators.

Molecular sequence data were used to verify that moth larvae feeding on host leaves were those of the pollinating adults and to infer the phylogenetic placements of the two moth species and the two plant species, given that all or in large and taxonomically poorly known groups. The DNA data also permitted molecular clock-based estimates of divergence times for the plant and insect partners and thus to test whether they could have interacted over much of their evolution. Finally, we used GC-MS to assess whether the moths might find their oviposition sites (i.e., young leaves of the host) and adult foraging sites (i.e., the flowers of the host) by a specific odor. The chemical breakdown of the pollen walls was documented photographically, using freshly caught, instantly killed moths and moths caught at flowers but then held captive and starved for 2–3 days. The sporopollenin-dissolving compound exuded by the moth proboscises could not be identified from dead moths, but studies of insect hemolymphs suggest at least one common candidate chemical.

## Results

### Plant and moth ecology and behavior

**Figure 1:**
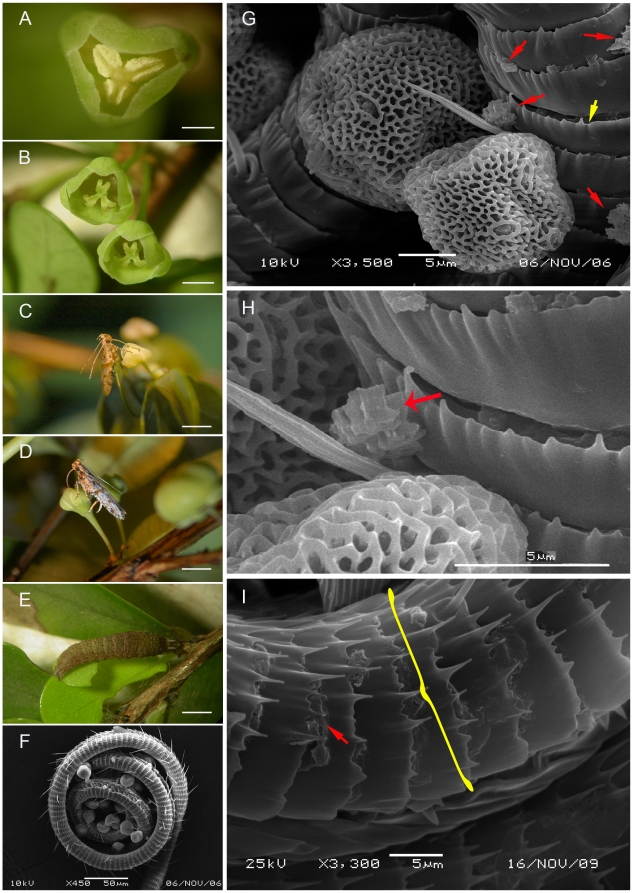
*Deltophora* sp. 1 moths and *Phyllanthus cochinchinensis* flowers and leaves photographed in the field (A–E) or under the SEM (F–I). A. Male flower with dehisced anthers. B. Female flowers with the bifid stigmas. C. *Deltophora* sp. 1 moth drinking nectar from a male flower. D. *Deltophora* sp. 1 moth drinking nectar from a female flower. E. Larva of *Deltophora* sp. 1 on a young leaf, the characteristic feeding damage is visible along the lower leaf margin. F. Pollen-coated proboscis from an instantly killed *Deltophora* sp. 1 moth; note that all pollen grains are intact. G. Intact and broken *P. cochinchinensis* pollen grains on a proboscis. Red arrows indicate exine fragments. The yellow arrow points to one of the rings (casings) sheathing the proboscis. H. An almost completely dissolved pollen wall (red arrow). I. The proboscis-surrounding casing (yellow bracket) of a moth killed following a few days of starvation. The red arrow points to a pollen wall fragment.

**Figure 2:**
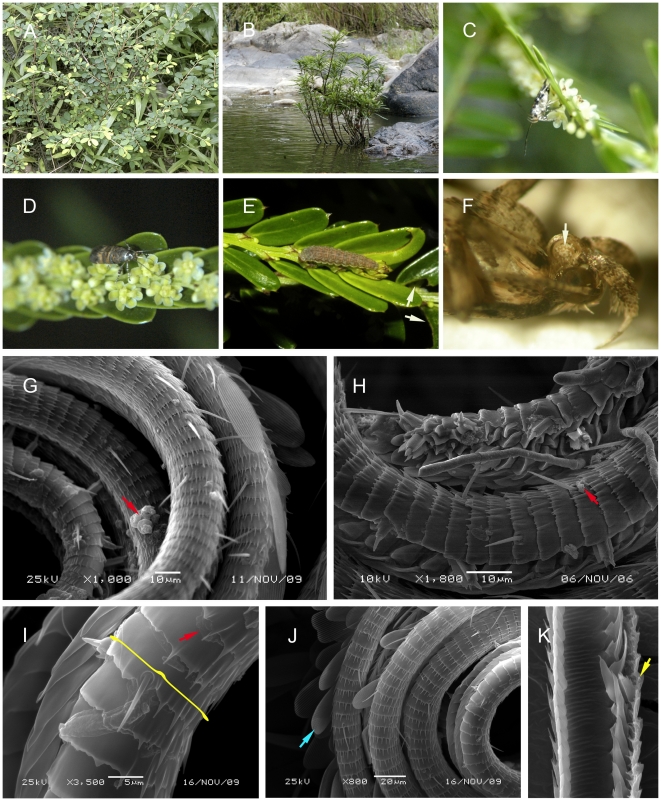
Habits of the focal plant species and *Deltophora* sp. 2 moths interacting with *Phyllanthus rheophyticus*. A Habit of *Phyllanthus cochinchinensis*. B. Habit of *P. rheophyticus*. C. *Deltophora* sp. 2 drinking nectar from a male flower of *P. rheophyticus*. D. *Deltophora* sp. 2 visiting a female flower of *P. rheophyticus*. E. Larva of *Deltophora* sp. 2 on young leaves of *P. rheophyticus*; the arrow points to the feeding damage. F. Pollen-coated recoiled proboscis of *Deltophora* (the arrow points to the pollen). G and H. Dissolved pollen wall fragments (red arrows) on the proboscis of a *Deltophora* moth killed after two days of starvation. I. Nearly dissolved sporopollenin (red arrow) and the unique integument casing (yellow brackets) on the proboscis of a *Deltophora* sp. 2 moth killed by starvation. J. The blue arrow marks one of the characteristic casings on the upper part of the proboscis of *Deltophora* sp. 2. K. Longitudinal section of a proboscis, showing the thick integument and the food canal through which nectar is sucked up. Dissolved pollen material might enter the food canal through the circular openings in the integument (yellow arrow).

During the 300 h of observations (at seven study sites in China and Vietnam; Materials and Methods), the only visitor and pollinator of *Phyllanthus cochinchinensis* was *Deltophora* sp. 1. During the 200 h of observations of the Hainan-endemic *P. rheophyticus*, the only flower visitor and pollinator was *Deltophora* sp. 2. No other insects visited the flowers. Beginning in the early evening and continuing until midnight, *Deltophora* moths would visit male flowers to forage for pollen and nectar, while female flowers were visited only for nectar (Fig. 1C and D; Fig. 2C and D). Moths switched between flowers and plants and were also observed laying their eggs on young leaves. Flower visits lasted 45–60 seconds (N = 10), with single flowers being probed about 10 times, meaning a moth would uncurl and curl its proboscis and insert it into the same flower (probing of male flowers 10.4±2.7 SD; probing of female flowers 9.6±2.3 SD; each with N = 5). Male and female moths carried pollen grains on their proboscises (Fig. 1F; Fig. 2F), and pollen grains under the microscope could unambiguously be identified as coming from the host plants. Cross-pollination is very effective as shown by the high fruit set in the dioecious species, *P. cochinchinensis* (Supporting Information S1). The male flowers typically produce 8400±2141 pollen grains (data for *P. cochinchinensis*), and the pollen of both *Phyllanthus* species is rich in lipids but lacks starch.

### Pollen walls dissolved by a secretion from the *Deltophora* proboscises

The proboscises of instantly killed *Deltophora* were c. 3 mm long (N = 20 for sp. 1; both species had similar body and proboscis sizes) and were covered with pollen grains (Fig. 1F; 2F). Their structure appears to be unique in Gelechioidea [18; L. Kaila, Senior Curator, Finnish Museum of Natural History, personal communication, 11 Nov. 2009]: It consists of curved integument casings that surround the proboscis (Fig. 1G–I; Fig. 2G–K). Remains of partially dissolved pollen grain walls on the proboscises of freshly killed moths (N = 40) are marked by red arrows in Fig. 1G–I as well as Fig. 2G–I. Moths caught on flowers (23 moths from *P. cochinchinensis* and 21 from *P. rheophyticus*) and then starved for 2–3 days inside large plastic bottles no longer had any intact grains on their proboscises. Pollen fragments lying between the circular integument casings suggest that the sporopollenin-dissolving chemical is produced between these casings. Older *Deltophora* specimens in our collection have brittle proboscises, perhaps because the corrosive action of this chemical attacks the proboscis over time, in spite of its strongly armored surface.

### Temporal fit between moth and plant life cycles

The life cycles of both moth species were closely linked to the phenology of their plant hosts, which appear to be the moths' only larval food plants. Experimental rearing of moth larvae in 2006, 2007, and 2008 succeeded on their host's leaves. Adult *Deltophora* appeared in mid-May and immediately mated. During May, June, and July, moths could regularly be seen visiting flowers. No adult moths were found before or after the hosts' flowering seasons. The youngest instar larvae fed internally on young leaves before emerging on the lower leaf surface. Older caterpillars reached 8–10 mm in length (Fig. 1E and 2E) and also fed only on young leaves. Larvae fed only at night; during the day they rested on lower leaf surfaces. The characteristic feeding damage is shown in Fig. 1E and Fig. 2E and the extent of leaf damage in Supporting Information S1.

Caterpillars of *Deltophora* sp. 1 placed on the leaves of *Glochidion eriocarpum* (Phyllanthaceae) did not feed. On their host leaves, however, larvae developed and pupated normally and eclosed after 30 to 36 days. A few instead entered a 9–11 month-long pupal diapause (under laboratory conditions) from which adults emerged simultaneous with the next flowering period of their plant host.

### Molecular phylogenetics and divergence time estimates for the plant and moth species

**Figure 3:**
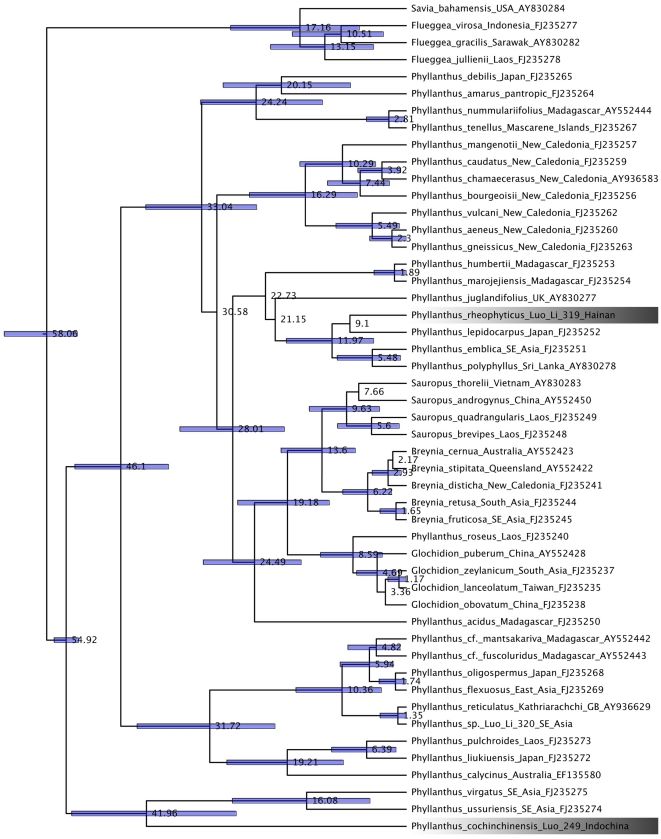
Chronogram for the Phyllantheae obtained under a Bayesian relaxed clock. Bars around node ages indicate the 95% highest posterior density intervals for nodes with a posterior probability >0.7. *Phyllanthus cochinchinensis* and *P. rheophyticus*, the species pollinated by the *Deltophora* moths studied here, are highlighted.

Of the c. 1270 described species of *Phyllanthus*, 130 have been sequenced for *matK*. In a maximum likelihood tree from these sequences, the two *Phyllanthus* species studied here are only distantly related, and a relaxed molecular clock calibrated with *Phyllanthus* fossils suggests that their most recent common ancestor lived 55 (38–54) Ma ago (Fig. 3).

**Figure 4:**
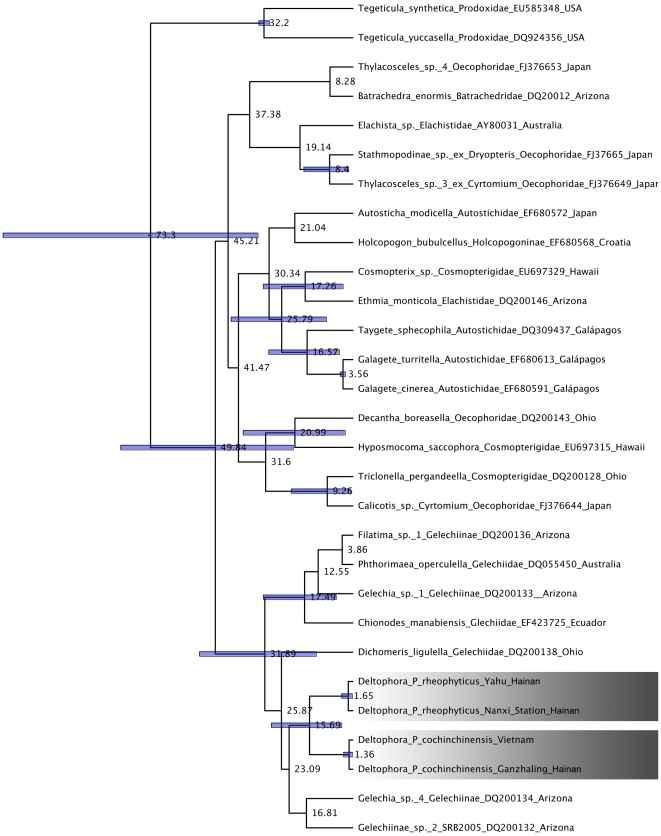
Chronogram for the *Deltophora* species feeding on *Phyllanthus cochinchinensis* and *P. rheophyticus* obtained under a Bayesian relaxed clock with lognormally distributed rates. Bars around node ages indicate the 95% highest posterior density intervals for nodes with a posterior probability >0.7. The distance between the study sites on Hainan Island and those in Vietnam is about 500 km, that between Yahu and Nanxi Station about 60 km.

For the moths, a maximum likelihood tree of 29 cytochrome c oxidase subunit I (COI) sequences, including sequences of *Tegeticula* and *Galagete* for calibration purposes, other Gelechioidea, and four sequences representing the two *Deltophora* species, showed that the studied species clearly belong in the Gelechiidae and that the larvae represented the same species as the adult moths (larval sequences are not included in Fig. 4 because redundant sequences were removed before the clock analyses; Materials and Methods). A relaxed clock model (Fig. 4) suggested a divergence time for the *Deltophora* species of 16 (29–4) Ma. This estimate is highly preliminary because of the extreme molecular under-sampling of Gelechioidea and the great genetic distances of the two calibration points. Nevertheless, the distant phylogenetic relationships of the two *Phyllanthus* species and the much younger divergence time of the moths (Figs. 3 and 4) together reject a hypothesis of co-speciation.

### Results of the GC-MS analyses

Details of the GC-MS method are given in Materials and Methods. The dominant compound detected in fresh *P. cochinchinensis* flowers and leaves was 1,3,6-octatriene (also called cis-β-ocimene). Other compounds were present in only small amounts and could not be identified. No unusual compounds were present in methanol or chloroform extracts of moth proboscises.

## Discussion

*Phyllanthus cochinchinensis* and *Deltophora* sp. 1, and *P. rheophyticus* and *Deltophora* sp. 2 are two pairs of obligate mutualists. No other insects visited the minute flowers during several hundred hours of observations, and the moths' entire life cycle, from the larvae feeding on the young leaves of their hosts to the adults emerging synchronously with flowering in the hosts, is adapted to their host plants' phenology and chemistry. That cis-β-ocimene is the dominant scent compound in both, the leaves and the flowers (of *P. cochinchinensis*; the other species was not analyzed), suggests that this is the key signal for host selection. Cis-β-ocimene has also been found in the female flowers of the Phyllanthaceae *Breynia vitis-idaea* [19], and is common in floral scents.

The genus *Deltophora* belongs to the Gelechiidae, a species-rich group of Lepidoptera that may not be monophyletic [20]. Most of the c. 870 genera of Gelechiidae are poorly defined, and the genus *Deltophora*, originally described from South Africa and supposedly containing 19 species on all continents except Antarctica, is no exception [21]. Prior to our observations, host plants of larvae and adults were unknown [21]. Without denser species sampling, it is not possible to infer how long the *Deltophora* moths may have interacted with their current *Phyllanthus* hosts, which are unexpectedly distantly related to each other as shown by the molecular phylogeny (Fig. 3).

This is the first case of an animal chemically dissolving spore walls, i.e., the sporopollenin in the pollen exine. Among the few substances known to dissolve sporopollenin is ethanolamine, also called 2-aminoethanol or monoethanolamine, which palynologists use to remove sporopollenin [12], [13]. Ethanolamine is an extremely volatile organic compound. It is an abundant head group for phospholipids, substances found in biological membranes, and is present in insect hemolymphs, usually as phosphatidyl-ethanol-amine. Ethanolamine is among the defense secretions exuded as droplets from glandular hairs by the pupa of the Mexican bean beetle, *Epilachna varivestis* [22], [23]. Given its known ability to dissolve sporopollenin, ethanolamine is a possible candidate for the unknown chemical produced by *Deltophora*. Chemical investigation of the compounds responsible for the spore wall breakdown will require the GC-MS analysis of freshly killed, perhaps even living moths; our analyses using methanol or chloroform extracts of several month-old proboscises yielded no unusual compounds.

Pollen feeding in other groups of Lepidoptera is entirely different than that in the gelechiid *Deltophora* studied here. Thus, *Heliconius* (including *Laparus*), a group of nymphalid Lepidoptera, produce saliva that has proteolytic activity [10], [24], and light microscopy has revealed that pollen grains exposed to the saliva for >200 min had modified “exines,” which has been attributed to proteolysis [25]. Sporopollenin, however, does not consist of proteins (nor do other spore wall layers). The proboscises of *Heliconius* butterflies bear long sensory bristles on which pollen is accumulated [7], [8]. They are entirely different from those of the *Deltophora* moths studied here. Just like these moths, however, *Heliconius* repeatedly insert and retract their proboscis when visiting single flowers, and it was this behavior that first alerted us to the moth's active up-take of pollen grains, not just nectar from the *Phyllanthus* flowers. The *Deltophora* larval stage lasts 30–36 days, which is relatively short; we do not know whether the moth's diet is supplemented with compounds from the pollen walls, but this could be tested with radioactive labeling.

The discovery of two new obligate plant/pollinator interactions, involving the hereto unknown complete breakdown of spore walls, shows how little is known about arthropod chemical interactions with their environment. Given this limited knowledge and the ubiquity of spore walls in nature, it seems unlikely that *Deltophora* moths should be the only arthropods capable of dissolving sporopollenin.

## Materials and Methods

### Plant species, study sites, and study duration

*Phyllanthus cochinchinensis* (Loureiro) Sprengel (vouchers Luo 248 and 249, deposited in the herbaria IBSC and M) is a shrub (Fig. 2A) that occurs in India, China, Cambodia, Laos, and Vietnam, usually in low vegetation or along forest edges at 200–600 m alt. The species is dioecious, with axillary inflorescences. Male flowers are borne in fascicles, but usually open one at a time. Each has three stamens that dehisce longitudinally (Fig. 1A). Female flowers are also borne in fascicles and have three bifid recurved styles (Fig. 1B). Both sexes offer nectar. *Phyllanthus cochinchinensis* was studied at seven sites in China and Vietnam (precise localities and duration of observations are given in the Supporting Information S1). Study sites were 7 to 800 km apart from each other.

*Phyllanthus rheophyticus* M. G. Gilbert & P. T. Li also is a small shrub, but different from *P. cochinchinensis* it is monoecious. The species is endemic to Hainan Island [26] where is grows along riverbeds (Fig. 2B). The axillary bisexual inflorescences usually produce two to four male flowers and one female flower. Flowers are similar to those of *P*. *cochinchinensis,* and again both sexes offer nectar. *Phyllanthus rheophyticus* was studied at three sites on Hainan Island.

From 2006 to 2010, observations where carried out between May and September each year, which are the only months during which the two *Phyllanthus* species produce flowers. Overall, flower visitors and leaf feeding larvae were studied for c. 300 h in *P. cochinchinensis* and for c. 200 h in *P. rheophyticus*, mostly at night but occasionally also during the day. At two study sites, flowering shrubs of *P. cochinchinensis* (30 staminate and 25 pistillate individuals) and *P. rheophyticus* (30 monoecious individuals) were monitored weekly during the entire reproductive season from May to September.

Nectar production was assessed both, during the day and at night, using 10 marked flowers of each sex on five plants of each species. Marked flowers were covered with fine netting to exclude nectar foragers, and nectar was sampled at 0800 h, 1200 h, 1600 h, 1900 h, 2100 h, and 2300 h. Pollen grains were tested for the presence of lipids or starch by staining in either sudan III or iodine (IKI) solutions, followed by examination under a microscope. Dimethylthiazol-diphenyl-tetrazolium bromide (MTT) was used to assess stigma receptivity. Experimental investigation of the plants' mating systems is described in the Supporting Information S1.

### Moth species and their behavior

Moths that visited the plants' flowers or leaves were collected for identification and examination of their pollen loads under a stereoscope (Stemi DV4; Zeiss, Germany). The proboscises of moths that were either killed immediately at flowers or following 2–3 days of captivity in large plastic bottles were examined under an SEM (JSM-6360LV, JEOL, Japan). We also searched for *Deltophora* adults or larvae outside the flowering seasons of their *Phyllanthus* hosts, checking especially co-occurring Phyllanthaceae, such as *Breynia fruticosa* (Müll.Arg.) Hook.f., *Glochidion eriocarpum* Zipp. ex Span., *Phyllanthus reticulatus* Poir., and *P. nanellus* P.T. Li. Adults and larvae were sent to specialists for identification (Acknowledgments), and vouchers have been deposited in the zoological collections of Nankai University and the Zoologische Staatssammlung in Munich (ZSM).

Male and female flowers visited by *Deltophora* moths were examined for moth eggs under a stereoscope. Both *Phyllanthus* species were regularly checked for moth eggs on leaves, inflorescences, and flower bracts, and moth larvae were brought to the laboratory for rearing on host leaves to study the length of larval and diapause stages. We also tried whether they would feed on *Glochidion eriocarpum* leaves. Numbers of lab-reared larvae in 2006, 2007, and 2008 are specified in the Supporting Information S1, and the quantification of leaf damage due to larval feeding is described in Supporting Information S1.

### Molecular phylogenetics and molecular clock-based divergence time estimation for the moths and their plant hosts

For molecular phylogenetics and molecular clock dating, total DNA was extracted from wild-caught and lab-reared *Deltophora* adults and larvae using standard methods. We PCR-amplified a part of the mitochondrial cytochrome oxidase subunit I gene (COI) using the primers CO1-F 5′-ATA ATT TTT TTT ATA GTT ATA C-3′ and CO1-R 5′-GAT GGG CTC ATA CAA TAA ATC CTA-3′ [4]. Sequences have been submitted to GenBank (GU247447-GU247452). *Deltophora* cytochrome c oxidase subunit I (COI) sequences were aligned with GenBank Gelechioidea sequences, with outgroup taxa chosen based on [18] and [27]. For time calibration (below), we included *Tegeticula* (Prodoxidae) and *Galagete* (Autostichidae). The tree was rooted on Prodoxidae.

For each *Phyllanthus* species, we isolated total genomic DNA from silica-dried leaves material using the methods described in [28] and sequenced the plastid maturase K (*matK*) gene for which over 130 other fully vouchered Phyllanthaceae sequences are available in GenBank [28]–[31]. The GenBank *matK* sequence of *Bridelia ferruginea* was renamed *Bridelia* sp. based on [28] and that of *Sauropus androgynus* became *S. garrettii* based on [32]. Primers were those described in [33]. New sequences have been submitted to GenBank (GU263803 and GU263804). The *matK* matrix used to infer closest relatives consisted of 132 sequences of Phyllanthaceae plus a few Picrodendraceae, Euphorbiaceae, and Putranjivaceae for rooting purposes.

Maximum likelihood (ML) analyses on the moth and plant matrices were performed under the GTR + G model of substitution using RAxML [34]. Bootstrap support was estimated in RAxML with 100 replicate heuristic searches under the same model as used in the searches. Maximum likelihood trees with branch lengths were then used in relaxed molecular clock models that incorporated at least one fossil-based calibration point (given as a probability distribution). Specifically, we used the uncorrelated-rates Bayesian relaxed clock approach implemented in BEAST v.1.4.8 [35], which uses a Markov chain Monte Carlo (MCMC) method to co-estimate topology, substitution rates, and node ages. Posterior probability distributions of node ages were obtained from the cytochrome c oxidase subunit I or *matK* alignments, with the number of taxa slightly reduced by taking out identical or near-identical moth and plant sequences. Analyses used a speciation model that followed a Yule tree prior, with rate variation across branches lognormally distributed. MCMC chains were run for 10 million generations, with parameters sampled every 1000^th^ step. Whether the chains had reached stationarity was checked using Tracer v. 1.4.1 [36]; this showed that the effective sample sizes for all estimated parameters were well above 200.

For calibration of the moth phylogeny, we used a normal prior distribution with a mean of 3.3 million years (Ma) and a SD of 1 Ma for the *Galagete* crown group and a mean of 32.4 Ma (SD 1 Ma) for the *Tegeticula* crown group. The divergence time between *Tegeticula synthetica* and *T. yuccasella* has been dated to 32.4 Ma [37]. The first radiation event on the Galápagos archipelago within the *Galagete* lineage has been dated to 3.3 Ma [27).

For calibration of the plant phylogeny, the crown group age of *Phyllanthus sensu lato* (including *Breynia*, *Glochidion*, and *Sauropus*) was set to 55 million years (Ma) (SD = 1 Ma) based on pollen described from the Lower Eocene Woolwich bed in Kent, England [38]. Although the pollen of *Phyllanthus* is heterogeneous, the Woolwich pollen appears to be correctly assigned. As an alternative minimal constraint, we could use fossil fruits that resemble those of *Phyllanthus* from the Maastrichtian, 65–70 Ma ago [39].

### GC-MS analyses of volatiles

Volatiles emitted from the young leaves and from female and male flowers were collected in the field by enclosing the leaves or clusters of 20–50 flowers for 2 hours within a polyacetate bag. Alternatively, 50 cut flowers were placed in 10 ml headspace glass vials (CNW Technologies GmbH, Düsseldorf, Germany) that were immediately sealed. We used solid phase microextraction (SPME; Sigma-Aldrich) and SPME fibre assemblies (65 µm PDMS/DVB, Sigma-Aldrich) to adsorb odour from the headspace. To analyze the proboscis chemistry of *Deltophora* sp. 1 moths, we placed up to ten proboscises in either methanol or chloroform solutions. Scent samples and proboscis extracts were analyzed by thermal desorbtion on a gas chromatograph/mass spectrometer (GC-MS) QP2010 Plus Shimadzu QP2010 instrument. The conditions used were column oven temperature 50.0°C, injection temperature 250.00°C, flow control mode linear velocity, pressure 53.5 kPa, total flow 12.0 mL/min, column flow 1.00 mL/min, linear velocity 36.3 cm/sec, and purge flow 6.0 mL/min. The GC-MS data were processed using GC Solution software release 2.5 SU3 (Shimadzu Co., Japan).

## Supporting Information

Supporting Information S1 Additional supporting information may be found in the online version of this article, specifically details on localities, observation times, quantification of leaf damage caused by the *Deltophora* larvae, and experiments on plant mating systems.
(DOC)
