# Supplementary material for: Gelechiidae Moths Are Capable of Chemically Dissolving the Pollen of Their Host Plants: First Documented Sporopollenin Breakdown by an Animal
Source: PLoS One. 2011 Apr 28;6(4):e19219. doi: 10.1371/journal.pone.0019219 (PMC3084278; doi:10.1371/journal.pone.0019219)
Supplement: Supporting Information S1 — Additional supporting information may be found in the online version of this article, specifically details on localities, observation times, quantification of leaf damage caused by the Deltophora larvae, and experiments on plant mating systems. (DOC) [file pone.0019219.s001.doc]

**Supporting information for “Gelechiidae Moths are Capable of Chemically Dissolving the Pollen of their Host Plants: First Documented Sporopollenin Breakdown by an Animal”**

**This file includes:**

Additional Materials and Methods

References for supplemental data

Tables S1 and S2

Additional Materials and Methods

***Study locations and observation times***

*P. cochinchinensis* was studied at seven sites in China and Vietnam (precise dates given below): (1) the grounds of the South China Botanical Garden, an area of 300 ha, including natural vegetation (SCBG), (2) Longyandong Forest Park (Longyandong), and (3) Shaojiwo Forest Park (Shaojiwo), all within the city area of Guangzhou; (4) Nankeshan Nature Reserve (Nankeshan), Zhaoqing City; and (5) Wuguishan Forest Park (Wuguishan), Zhongshan City, all in Guangdong Province; (6) Ganzhaling Nature Reserve (Ganzhaling), Sanya City on Hainan Island; and (7) near Honghai in Quangniah Province, Vietnam. *Phyllanthus rheophyticus* was studied in 2007 and 2008 at three sites, namely (1) Diaoluoshan Nature Reserve (Nanxi station and Nanxi village), (2) Wuzhishan Nature Reserve (Changhao Farm, Wuzhishan city), and (3) Yingouling Nature Reserve (Yahu Vilage, Maoyang Town) all on Hainan Island. The distances between these sites are: Honghai in Vietnam to Ganzhaling on Hainan Island 500 km; Honghai to SCBG or Longyandong 800 km; Honghai to Wuguishan Forest Park 700 km; SCBG or Longyandong to Ganzhaling 700 km; SCBG or Longyandong to Nankeshan 75 km; Ganzhaling to Wuzhishan 65 Km; Wuzhishan to Yahu 40 km, Wuzhishan to Diaoluoshan 50 km; Ganzhaling to Nanxi station or village about 70 km; Nanxi station to Nanxi village about 7 km, and Nanxi to Yahu 60 km.

In 2006, 2007, 2008, 2009, and 2010 we carried out nocturnal and some diurnal observations on flowering plants of the two species at the ten study locations as follows: Day observations for 4, 2, and 2 days at SCBG in 2006, 2007, 2008, and 2010 (July); 1 day at Longyandong in 2006; 1 day at Gangzhaling in 2007; 2 days at Diaoluoshan in 2007 and 2008; and 1 day at Yahu in 2007 and 2008. Night observations between 1900 and 0600 h from 10 May to 17 September, 2006 and 2008 and May–July, 2007, all at SCBG; 7 June, 27 September 2006 and 15–19 July 2007 at Longyandong; 4–7 August 2006 at Shaojiwo; 28–29 May 2007 at Wuguishan; 10-14 August 2007 at Gangzhaling; 26 September 2007 at Lankeshan; 16–17 August 2008 at Honghai; 4–6 June 2007, 25–27 June, and 2–4 August 2008 at Diaoluoshan (Nanxi Station and Nanxi Village); 2–3 June 2007, 28–29 June, and 5 August 2008 at Yahu; and 6–7 August 2008 at Changhao. Overall, moth pollination and leaf feeding were studied for over 300 h in *P. cochinchinensis* and for some 200 h in *P. rheophyticus*. In July 2010, we sampled the floral odor from *P. cochinchinensis* at SCBG and also collected adult moths for Y-tube choice experiments.

***Plant phenology***

Flowering of *P. cochinchinensis* (30 staminate and 25 pistillate individuals) and *P. rheophyticus* (30 monoecious individuals) was monitored weekly during the entire reproductive season from May to September, respectively, at SCBG and Diaoluoshan. Flowering dynamics were monitored on 10 male and female flowers from 10 plants of *P. cochinchinensis* and *P. rheophyticus* at SCBG and Diaoluoshan, using the following binning, (i) closed buds (petals still enclosed by the calyces), (ii) open flowers (pollen presentation of male flowers or female flowers with calyces spread opening), (iii) post-anthesis (male flowers turn yellow, onset of fruit development in female flowers). We also monitored leaf bud break, flushing, and shedding of individual leaves.

***Nectar production, pollen histochemistry, and experimental pollinations***

Nectar production was assessed both, during the day and at night, using 10 marked flowers of each sex on five plants. Marked flowers were covered with fine netting to exclude nectar foragers, and nectar was sampled at 0800 h, 1200 h, 1600h, 1900 h, 2100 h, and 2300 h. Presence of lipids or starch in pollen grains was tested with alternate staining in either sudan III or iodine (IKI) solutions, followed by examination under a microscope. Dimethylthiazol-diphenyl-tetrazolium bromide (MTT) was used to assess stigma receptivity (Rodriguez-Riaño and Dafni, 2000), and hand pollinations were carried out on flowers on five female plants at SCBG, and fruit set was simultaneously recorded on nearby controls.

***Plant phenology, nectar production, and fruit set from***

***natural and experimental pollinations***

In both *Phyllanthus* species, leaf buds and flower buds developed simultaneously, and from early/mid May through early October, tender young leaves were produced more or less continuously, simultaneous with the flowers. At the Chinese sites, tender young leaves were available from mid-May to late September, and flowering lasted from mid-May to mid-September. A single leaf reached its mature size within 7–10 days (N = 20), and during the following 6-10 days it stayed tender and light yellow-green (Fig. S1E). A single shrub typically produced young leaves for 1–3 months (early/mid May to late September). At SCBG, the entire population of *P.* *cochinchinesis* bloomed synchronously during the first part of the flowering season, with some plants then flowering a second or even a third time. 70% (21/30) of staminate individuals and 48% (12/25) of pistillate individuals flowered more than once; 16.6% (5/30) staminate and 8% (2/25) pistillate individuals flowered three times.

In both *Phyllanthus* species, flower development took about 10 days for male flowers, and about 12 days for female flowers. Flowers opened shortly after nightfall, and in staminate individuals, the anthers dehisced soon thereafter. Female flowers had receptive stigmas for 2–3 nights (based on the MTT test). Male flowers offered pollen for just one night (rarely 2 nights).

Nectar secretion lasted from about 1700 h until 0600 h (and throughout a flowers life span). Nectar volumes were minute. Staminate flowers of *P. cochinchinensis* produce 8400 ± 2141 pollen grains (SD, N = 5), and female flowers have 6 ovules (N = 20), yielding a P/O ratio of 1400 ± 357 (SD). *Phyllanthus cochinchinensis* and *P. rheophyticus* pollen is rich in lipids and contains no starch as assessed by Sudan III solution and IKI solution, respectively.

Hand-assisted cross-pollination resulted in a fruit set of 74 ± 8 % in *P. cochinchinensis*, compared with a fruit set of 63 ± 13 % from open pollination (Table 2). Bagged (netted) flowers of either species set no fruit, indicating the absence of agamospermy. Fruits reached maturity within three weeks, and there was no sign of insect damage (*P. cochinchinensis*; N = 400 fruits at SCBG and Longyandong; *P. rheophyticus*, N = 260 fruits at Diaoluoshan).

Staminate flowers of *P. cochinchinensis* produce 8400 ± 2141 pollen grains (SD, N = 5), and female flowers have 6 ovules (N = 20), yielding a P/O ratio of 1400 ± 357 (SD). *Phyllanthus cochinchinensis* and *P. rheophyticus* pollen is rich in lipids and contains no starch as assessed by Sudan III solution and IKI solution, respectively.

***Quantification of moth feeding damage and moth rearing experiments***

*Deltophora* sp. 1 was never seen visiting the flowers or leaves of sympatric and simultaneously flowering *Breynia fruticosa*, *Glochidion eriocarpum*, or *Phyllanthus nanellus*, and no adults or larvae of *Deltophora* sp. 2 were seen on sympatrically flowering *P. nanellus* and *Phyllanthus* *reticulatus.* From 7 July to 9 September 2006, SXL and YQL collected 24 moth larvae at SCBG and Longyandong, all from young leaves; 14 of these were reared in the lab, 10 were preserved in 70% ethanol solution for later study. In 2007 larvae were again brought to the lab and most eclosed after 30 to 36 days (N = 7); seven eclosed 9–11 months later, in May 2008. On 25 September 2007, a few larvae were still present on young leaves, although no more adults could be found. In 2008, random observation on *P. cochinchinensis* at SCGB yielded 5, 12, 10, 16 and 6 caterpillars (all on young leaves) in June, July, August, September and October. At Gangzhaling, results were similar.

In the three *P. rheophyticus* populations, *Deltophora* sp. 2, larvae were found during most nights. On 2 and 3 June 2007, we collected 10 larvae at Yahu; during 4–6 June 2007, we collected 12 and 7 larvae at Nanxi Station and Nanxi village; on 25–29 June and 2–7 August 2008, 8 and 17 larvae were collected. Only during the earliest observation period, 25–27 April 2007, could we not find any larvae. In total, we reared 20 *Deltophora* sp. 2 in 2007 and 12 in 2008, which resulted in 18 adults.

Young leaves in *P.* *cochinchinensis* measure 8–16 mm in length, those of *P. rheophyticus* 4–9 mm. To quantify leaf damage due to larval feeding, we randomly sampled leaves on 5 branches from 9 to 20 individuals at nine study populations, counting intact and damaged young leaves. No moths fed on fruits or seeds.

**References for Supplemental Data**

Rodriguez-Riaño T., Dafni A. 2000. A new procedure to assess pollen viability. *Sexual Plant Reproduction* 12: 241–244.

Table S1. Feeding damage by *Deltophora* larvae on *Phyllanthus cochinchinensis* and *P. rheophyticus* leaves in the different study populations.

| Locality | Year (sex) | Young leaves examined (individuals) | Damaged  young leaves per branchlet  (mean ± SD) | Intact  young leaves per branchlet  (mean ± SD) | Percentage of damaged young leaves |
| --- | --- | --- | --- | --- | --- |
| *Phyllanthus cochinchinensis* | |  |  |  |  |
| SCBG | 2006 (male) | 885 (13) | 3.7 ± 2 | 9.9 ± 5 | 27 |
|  | 2006 (female) | 622 (10) | 3.1 ± 3 | 9.3 ± 4 | 25 |
| Longyandong | 2006 (male) | 590 (10) | 4.0 ± 3 | 7.8 ± 3 | 34 |
|  | 2006 (female) | 663 (12) | 3.0 ± 2 | 8.0 ± 3 | 28 |
| Lankeshan | 2007 (male) | 124 (16) | 3.6 ± 3 | 4.1 ± 2 | 47 |
|  | 2007 (female) | 90 (9) | 6.1 ± 2 | 3.9 ± 2 | 61 |
| Ganzhaling | 2007 (male) | 188 (15) | 3.8 ± 3 | 8.7 ± 4 | 30 |
|  | 2007 (female) | 171 (15) | 3.1 ± 2 | 8.3 ± 4 | 28 |
| Honghai | 2008 (male) | 280 (15) | 8.1 ± 3 | 11.2 ± 3 | 40 |
|  | 2008 (female) | 315 (15) | 6.1 ± 2 | 14.3 ±6 | 32 |
| *Phyllanthus rheophyticus* | |  |  |  |  |
| Nanxi Station | 2007 | 597 (15) | 3.1 ± 2 | 36.7 ± 7 | 8 |
| Nanxi village | 2007 | 554 (15) | 5.7 ± 4 | 30.5 ± 6 | 17 |
|  | 2008 | 514 (15) | 9.8 ± 6 | 24.5 ± 7 | 29 |
| Yahu village | 2007 | 332 (10) | 4.6 ± 2 | 26.6 ± 4 | 20 |
|  | 2008 | 317 (10) | 10.4 ±4 | 20.3 ± 4 | 36 |
| Changhao | 2008 | 656 (20) | 10.2 ±5 | 22.7 ± 7 | 31 |

Table S2. Fruit set in *Phyllanthus cochinchinensis* subject to three treatments at the South China Botanical Garden, mean ± SD.

| Treatment | Number of flowers | Number of fruits | Fruit set (%) |
| --- | --- | --- | --- |
| Open pollination | 74 (6) | 56 | 63 ± 13 |
| Assisted pollination | 44 (5) | 33 | 74 ± 8 |
| Bagged flowers | 62 (5) | 0 | 0 |
